# Protective Effects of Adrenomedullin on Rat Cerebral Tissue After Transient Bilateral Common Carotid Artery Occlusion and Reperfusion

**DOI:** 10.21470/1678-9741-2019-0059

**Published:** 2020

**Authors:** Mehmet Kirisci, Hakan Gunes, Aydemir Kocarslan, Tuba Ozcan Metin, Duygun Altintas Aykan, Muhammed Seyithanoglu, Adem Doganer, Gulsen Bayrak, Ekrem Aksu

**Affiliations:** 1 Department of Cardiovascular Surgery, Faculty of Medicine, Kahramanmaraş Sütçü İmam University, Kahramanmaraş, Turkey.; 2 Department of Cardiology, Faculty of Medicine, Kahramanmaraş Sütçü İmam University, Kahramanmaraş, Turkey.; 3 Department of Histology and Embryology, Faculty of Medicine, Kahramanmaraş Sütçü İmam University, Kahramanmaraş, Turkey.; 4 Department of Pharmacology, Faculty of Medicine, Kahramanmaraş Sütçü İmam University, Kahramanmaraş, Turkey.; 5 Department of Biochemistry, Faculty of Medicine, Kahramanmaraş Sütçü İmam University, Kahramanmaraş, Turkey.; 6 Department of Biostatistics, Faculty of Medicine, Kahramanmaraş Sütçü İmam University, Kahramanmaraş, Turkey.; 7 Department of Histology and Embryology, Faculty of Medicine, Mersin University, Mersin, Turkey.

**Keywords:** Carotid Artery, Common, Neurons, Brain Ischemia, Adrenomedullin, Malondualdehyde, Reperfusion Injury, Glutathione Peroxidade, Rats

## Abstract

**Objective:**

We aimed to investigate the protective effect of adrenomedullin (ADM) on cerebral tissue of rats with cerebral ischemia/reperfusion (I/R) injury.

**Methods:**

Thirty-two Wistar rats were randomized into four groups (n=8). In the I/R Group, bilateral common carotid arteries were clamped for 30 minutes and, subsequently, reperfused for 120 minutes. In the ADM Group, rats received 12 µg/kg of ADM. In the I/R+ADM Group, bilateral common carotid arteries were clamped for 30 minutes and, subsequently, the rats received 12 µg/ kg of ADM. Then, reperfusion was performed for 120 minutes. The Control Group underwent no procedure. Blood and brain tissue samples were collected for biochemical and histopathological analysis. Serum malondialdehyde (MDA), superoxide dismutase (SOD), and glutathione peroxidase (GPx) were analysed. Brain tissue was evaluated histopathologically and neuronal cells were counted in five different fields, at a magnification of ×400.

**Results:**

Brain MDA in I/R Group was significantly higher than in ADM Group. Brain GPx and SOD in I/R+ADM Group were significantly higher than in I/R Group. The number of neurons was decreased in I/R Group compared to the Control Group. The number of neurons in I/R+ADM Group was significantly higher than in I/R Group, and lower than in Control Group. Apoptotic changes decreased significantly in I/R+ADM Group and the cell structure was similar in morphology compared to the Control Group.

**Conclusion:**

We demonstrated the cerebral protective effect of ADM in the rat model of cerebral I/R injury after bilateral carotid artery occlusion.

**Table:** 

Abbreviations, acronyms & symbols				
**ADM**	**= Adrenomedullin**	** **	**M**	**= Molars**
**COX-2**	**= Cyclooxygenase-2**		**MDA**	**= Malondialdehyde**
**DTT**	**= Dithiothreitol**		**NADPH**	**= Nicotinamide adenine dinucleotide phosphate**
**EO**	**= Essential oil**		**ROS**	**= Reactive oxygen species**
**GPx**	**= Glutathione peroxidase**		**SD**	**= Standard deviation**
**I/R**	**= Ischemia/reperfusion**		**SOD**	**= Superoxide dismutase**
**IL**	**= Interleukin**		**TOS**	**= Total oxidant status**
**IMA**	**= Ischemia-modified albumin**			

## INTRODUCTION

Carotid artery disease is the major risk factor for ischemic stroke^[1]^. In the treatment of ischemic stroke, the present guidelines mainly recommend the administration of tissue plasminogen activator as intravenous thrombolysis and mechanical thrombectomy^[2]^. The clinical team and the infrastructure that will successfully perform the mechanical thrombectomy procedure are not available in every hospital. Brain-protective agents used in clinical practice are not ideal preservatives. Therefore, it is very important to strengthen the research of new brain-protective agents and to seek new treatment options. Revascularization of occluded vessels will cause cerebral ischemia/reperfusion (I/R) injury in both stroke patients and experimental models^[3]^. During I/R, at the end of the ischemic process, an inflammatory response occurs by reperfusion of the tissues. Due to inflammation, extensive microvascular dysfunction and changes in tissue barrier functions may occur. Reactive oxygen species (ROS) occuring in the reoxygenated tissues cause deoxyribonucleic acid, protein, and plasma membrane injury^[4]^. In the formation of cerebral I/R injury, excitotoxicity, free oxygen radical release, inflammatory responses, reduction in energy supply, and many different mechanisms, such as ischemic apoptosis or necrosis, play a role^[5-7]^.

Adrenomedullin (ADM) is a strong vasodilator peptide isolated from human pheochromocytoma, which was first described in 1993^[8]^. ADM is produced only in the adrenal medulla, at the same time in the lungs, heart, kidney, and endothelial and vascular smooth muscle cells^[9]^.ADM has been involved in various biologic activities such as vasodilation, bronchodilation, neurotransmission, neuromodulation, and prevention of apoptosis. It is also known as an anti-inflammatory, antioxidative, antimicrobial, and antiapopitotic agent, and it has important roles in cardiovascular hemostasis, development of cardiovascular tissue and bone, regulation of hormone secretion, balancing of body fluid and electrolyte balance, and angiogenesis^[10,11]^.

The aim of this study was to investigate the protective effect of ADM against cerebral İ/R injury induced by bilateral carotid artery occlusion in rats.

## METHODS

A total of 32 adult Wistar-albino rats, weighing 200-250 g, were used in the study. The rats were housed and maintained at 22 °C, 60±5% humidity, and a 12:12 hours light/dark cycle, with free access to food and water *ad libitum*. After the study was approved by the animal ethics committee of Kahramanmaraş Sütçü İmam University (approval date: March 20, 2018; number: 4), it was carried out in the Kahramanmaraş Sütçü İmam University animal experiments laboratory. All experimental procedures were performed according to the principles of laboratory animal care and use.

### Experimental Design

All rats in the study were anesthetized with ketamine hydrochloride (Ketalar, 50 mg/kg, intramuscularly, Parquet-Davis, Eczacıbaşı, İstanbul, Turkey). Additional ketamine hydrochloride was administered intramuscularly (25 mg/kg) for anesthesia during the procedure. Surgical procedures were performed after the neck region of the rats was shaved and cleaned aseptically in the supine position under the heating lamp. The trachea was cannulated and artificial respiration was applied. Rats were randomized into four groups (n=8/group): Control Group, ADM Group, I/R Group, and IR+ADM Group. In the Control Group, an incision was made from the mid-neck region, the bilateral common carotid artery was found and dissected from the surrounding tissue, and the incision was closed. The ADM Group was administered intraperitoneally 12 µg/kg of ADM (Sigma, St. Louis, Missouri, United States of America); an ıncision was made in the middle of the neck, the bilateral common carotid artery was found and dissected from the surrounding tissue, and the incision was closed. In I/R Group, an incision was made in the mid-neck region, the bilateral common carotid artery was found and dissected from the surrounding tissue, and the bilateral carotid artery was clamped with nontraumatic microvascular clamp. Distal flow was observed and no flow was detected after clamping. After 30 minutes of ischemia, the microvascular clamp was removed and reperfused for 120 minutes. In I/R+ADM Group, an incision was made in the mid-neck region, the bilateral common carotid artery was found and dissected from the surrounding tissue, and the bilateral carotid artery was clamped with non-traumatic microvascular clamp. Distal flow was observed and no flow was detected after clamping. After 30 minutes of ischemia and at the end of the ischemia period, 12 µg/kg of ADM was given intraperitoneally as bolus. At the end of the ischemic period, the cross-clamps were removed and the cerebral tissue was reperfused for 120 minutes. At the end of the reperfusion period, all rats were sacrificed under anesthesia and serum and brain samples were collected and stored for biochemical and histopathological analysis. ADM administration doses were decided according to previous studies of Dwivedi et al.^[12]^.

### Histopathological Examination

The brain tissues obtained from each animal were immediately fixed in 10% neutral-buffered formalin for 48 hours, dehydrated through a graded series of ethanol, then cleared with xylol and embedded in paraffin blocks. Sections of 5 µm were obtained and stained with hematoxylin and eosin and cresyl violet for histomorphological evaluations. Stained sections were examined under a light microscope (Olympus BX-50; Olympus GmbH, Hamburg, Germany), and photographs were obtained for all groups. The assessment of the brain tissue was done based on previous study^[13]^. Two sections per animal were choosen to be evaluated. Five fields of the cortex in each section were randomly selected and the number of intact and damaged neurons showing the features of ischemic cell change (shrunken cell bodies, pyknotic nuclei, and eosinophilic cytoplasm) were counted at a magnification of x400 by an observer who was blinded to the groups.

### Biochemical Examination

At the end of the experimental procedure, all blood samples taken from the rats sacrificed were centrifuged for 10 minutes at 3000 revolutions per minute and the plasmas were separated. The plasmas were stored at -80 °C until analysis. Cerebral tissue samples obtained after the experiment were stored at -80 °C until biochemical analysis was performed. Glutathione peroxidase (GPx), superoxide dismutase (SOD), malondialdehyde (MDA), and ischemia-modified albumin (IMA), which are indicators of oxidative stress, were studied in cerebral tissue samples and blood serum. On the day of analysis, the samples were thawed. Cerebral tissues were homogenized with ice-cold 0.15 molars (M) of potassium chloride (10%, w/v). Tissue homogenates were centrifuged at 600 x g for 10 minutes at 4 °C to remove the crude fractions. Subsequently, the supernatants were centrifuged at 10,000 x g for 20 minutes to obtain the postmitochondrial fraction.

SOD and GPx activities were determined in postmitocondrial fraction. MDA levels were determined in tissue homogenates. SOD and GPx activities and MDA and IMA levels were measured in the obtained serums.

Serum and tissue MDA levels were assayed using thiobarbituric acid according to the method of Buege and Aust^[14]^. Distilled water (0.5 mL) was added to tubes containing 0.5 mL of serum or tissue homogenate. Buege separator (2 mL) was added (15 w/v trichloroacetic acid, 0.375 w/v% thiobarbuturic acid, and 0.25 mol/l hydrochloric acid solution mixed in equal volumes) to the mixture. The tubes containing the mixture were boiled in a boiling water bath for 15 minutes. After cooling, the precipitate was centrifuged at 4000 rpm for 10 minutes. The absorbance of the samples was determined by spectrophotometric method at 535 nm.

SOD activity was measured by the method described by Beyer and Fridovich. In this method, xanthine and xanthine oxidase are used to form superoxide radicals that react with 2-(4-iodophenyl)-3-(4-nitrophenyl)-5-phenyl-2*H*-tetrazolium chloride to form the red formazone dye. SOD activity is then measured by the degree of inhibition of this reaction^[15]^.

GPx activity was measured using the method of Paglia and Valentine with cumene hydroperoxide as substrate. In this method, GPx activity was coupled to the oxidation of nicotinamide adenine dinucleotide phosphate (NADPH) by glutathione reductase, and the oxidation of NADPH was followed spectrophotometrically at 340 nm, at 37 °C. Results were calculated using extinction coefficient (6.22 × 103/M cm)^[16]^.

Serum IMA levels were measured spectrophotometrically by the method developed colorimetrically by Bar et al.^[17]^. First, 10 µl of 1 g/l cobalt chloride solution was added to 40 µl of the serum, mixed, and allowed to incubate for 10 minutes at room temperature. Then, 10 µl of a 1.5 g/l dithiothreitol (DTT) solution was added and mixed, and it was ıncubated for two more minutes at room temperature. And finally, 200 µl of a 9.0 g/l solution of sodium chloride was added. Blind specimen were similarly prepared without the addition of DTT. The absorbances of the test mixtures were detected at 470 nm. The results were evaluated in absorbance units.

### Statistical Analyses

In the data evaluation, the conformity of the variables to the normal distribution was examined with Shapiro-Wilk test. The difference between groups in normal distribution variables was examined by one-way analysis of variance, or ANOVA. Tukey’s HSD, Tamhane’s T2, and Dunnett tests were used as *post hoc* tests (multiple comparison). The statistical parameters are expressed as mean±standard deviation. The findings are supported by tables and graphs. Data were analyzed with IBM SPSS Statistics, version 22 (IBM SPSS for Windows, version 22, IBM Corporation, Armonk, New York, United States of America).

## RESULTS

When brain tissue and blood serum results were evaluated, there were significant differences between groups in brain tissue results (Table 1), but there was no significant difference in serum results (Table 2). All rats were followed closely during the experiment. No complication nor death of animals were observed during the experiment.

**Table 1:** Biochemical assessment of brain tissue.

	Control (n=8)	ADM (n=8)	I/R (n=8)	I/R+ADM (n=8)		
	Mean±SD	Mean±SD	Mean±SD	Mean±SD	F	*P*-value
MDA (nmol/mL)	54.95±5.40	51.35±5.86^b^	63.15±1.61^a^	57.36±6.15	4.725	0.013*
GPx (nmol/mL)	24.24±3.89	22.44±1.33	20.45±1.89^c^	28.69±4.87^b^	6.130	0.005*
SOD (U/mL)	9.79±1.05	9.00±0.90	8.04±1.05^c^	10.52±1.63^b^	3.917	0.028*

* The difference is statistically significant;

a the difference from the adrenomedullin (ADM) Group was statistically significant;

b the difference from the ischemia/reperfusion (I/R) Group was statistically significant;

c the difference from the I/R+ADM Group was statistically significant. Different letters denote significant differences (*P*<0.05).

GPx=glutathione peroxidase; MDA=malondialdehyde; SD=standard deviation; SOD=superoxide dismutas

**Table 2:** Biochemical assessment of blood serum.

	Control (n=8)	ADM (n=8)	I/R (n=8)	I/R+ADM (n=8)		
	Mean±SD	Mean±SD	Mean±SD	Mean±SD	F	*P*-value
GPx (nmol/mL)	96.26±2.66	96.56±3.45	96.04±3.97	97.71±3.41	0.317	0.871
MDA (nmol/mL)	24.52±1.61	28.33±4.07	25.05±4.49	19.79±7.41	2.179	0.133
SOD (U/mL)	1.82±0.27	1.87±0.18	1.90±0.16	2.07±0.46	0.688	0.571
IMA (absorbance units)	0.31±0.05	0.29±0.10	0.33±0.05	0.33±0.08	0.338	0.798

ADM=adrenomedullin; GPx=glutathione peroxidase; IMA=ischemia-modified albumin; I/R=ischemia/reperfusion; MDA=malondialdehyde; SD=standard deviation; SOD=superoxide dismutase

### Serum Biochemistry Results

There was no significant difference between the groups regarding serum GPx values (*P*=0.871); Control Group (96.26±2.66), ADM Group (96.56±3.45), I/R Group (96.04±3.97), and I/R+ADM Group (97.71±3.41) (Table 2).

There was no significant difference in serum MDA levels between the groups (*P*=0.133); Control Group (24.52±1.61), ADM Group (28.33±4.07), I/R Group (25.05±4.49), and I/R+ADM Group (19.79±7.41) (Table 2).

Serum SOD values were not significantly different between the groups (*P*=0.571); Control Group (1.82±0.27), ADM Group (1.87±0.18), I/R Group (1.90±0.16), and I/R+ADM Group (2.07±0.46) (Table 2).

Serum IMA values were not significantly different between the groups (*P*=0.798); Control Group (0.31±0.05), ADM Group (0.29±0.10), I/R Group (0.33±0.05), and I/R+ADM Group (0.33±0.08) (Table 2).

### Tissue Biochemistry Results

When tissue MDA values were compared between the groups, I/R Group values (63.15±1.61) were statistically higher than ADM Group values (51.35±5.86) (*P*<0.05), and there was no significant difference between other groups (Table 1).

There was a significant difference between I/R Group values (20.45±1.89) and I/R+ADM Group values (28.69±4.87) when GPx values in brain tissue were compared (Table 1). I/R+ADM Group values were significantly higher compared to I/R Group values (*P*<0.05).

When tissue SOD values were evaluated, there was a significant difference between I/R Group values (8.04±1.05) and I/R+ADM Group values (10.52±1.63) (Table 1). I/R+ADM Group values were significantly higher compared to I/R Group values (*P*<0.05).

### Histopathologic Results

**Fig. 1:**
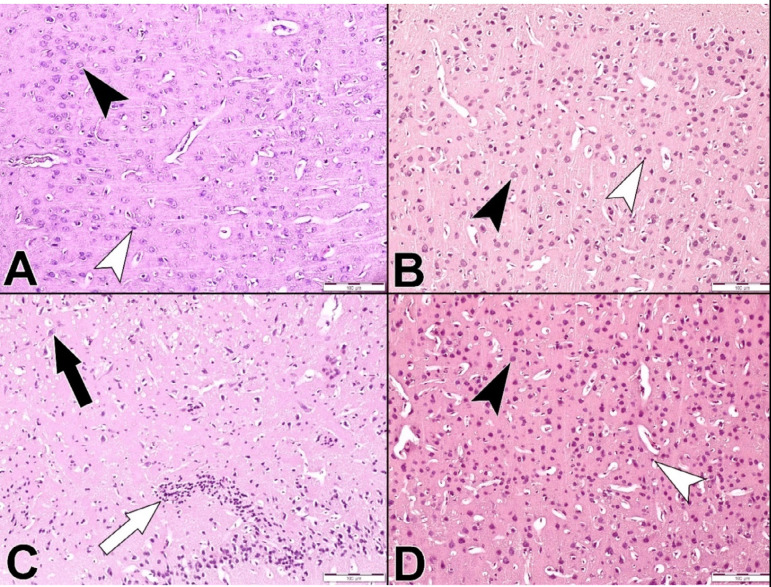
Hematoxylin and eosin staining. Control Group (A), adrenomedullin (ADM) Group (B), ischemia/reperfusion (I/R) Group (C), and I/R+ADM Group (D). Neurons (black arrowhead) and glial cells (white arrowhead), pyknotic nuclei (black arrow) and gliosis (white arrow). ´100.

**Fig. 2:**
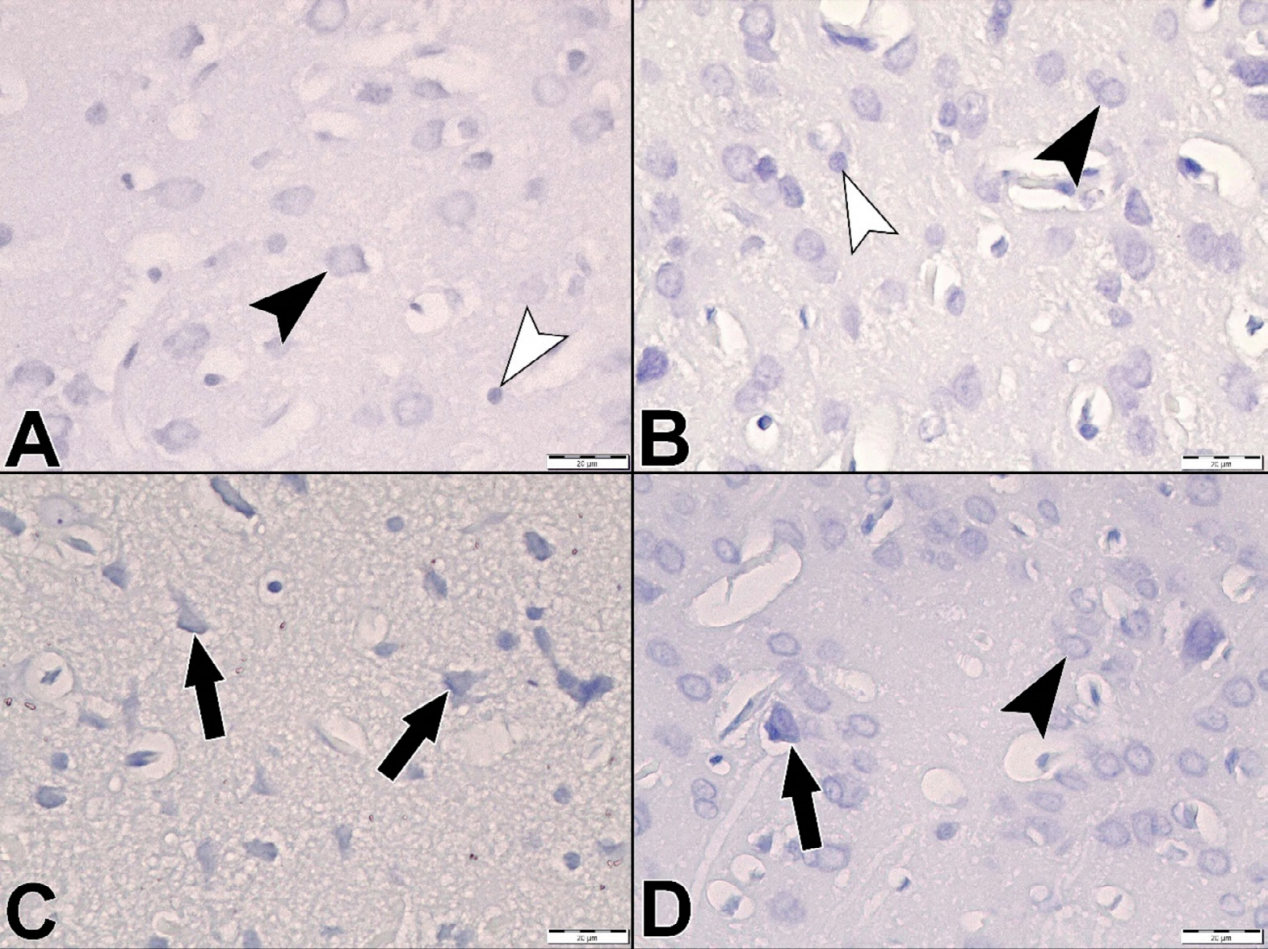
Cresyl violet staining. Control Group (A), adrenomedullin (ADM) Group (B), ischemia/reperfusion (I/R) Group (C), I/R+ADM Group (D). Neurons (black arrowhead) and glial cells (white arrowhead), pyknotic nuclei (black arrow). ´400.

The brain tissues obtained from the control and drug groups showed normal histological architecture (Figure 1A and B). I/R Group showed shrunken cell bodies, pyknotic nuclei, eosinophilic cytoplasm, and multiple focal areas of gliosis (Figure 1C). The I/R+ADM Group showed marked reduction of damaged neuron (Figure 1D). The number of neuron that were stained with cresyl violet and counted from different sections were examined (Figure 2).

**Fig. 3:**
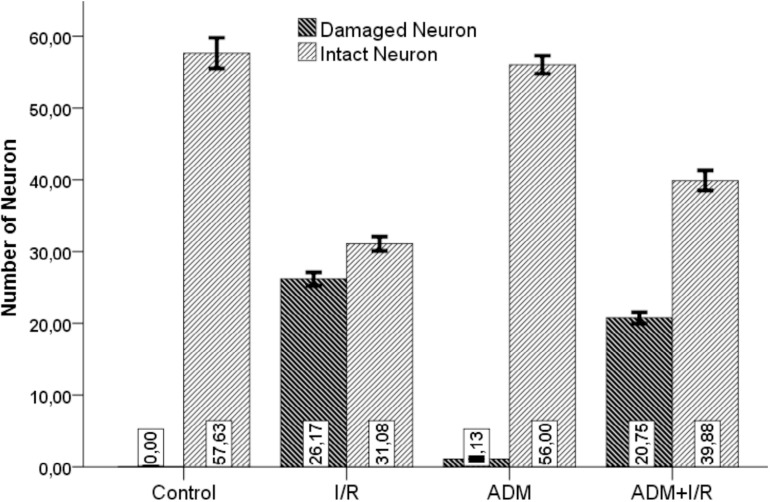
Comparison of number of brain neurons between groups. ADM=adrenomedullin; I/R=ischemia/reperfusion

In I/R Group, there was a statistically significant decrease in the number of intact neurons compared to the Control Group after İ/R (*P*<0.05). Administration of ADM reduced the number of damaged neurons and markedly increased the number of intact neurons (*P*<0.05) (Figure 3 and Table 3).

**Table 3:** Number of brain tissue neurons.

	Control (n=8)	ADM (n=8)	I/R (n=8)	I/R+ADM (n=8)		
Number of neurons	Mean±SD	Mean±SD	Mean±SD	Mean±SD	F	*P*-value
Damaged neurons	0.00±0.00^c,d^	1.13±0.64^c,d^	26.17±3.30^a,b,d^	20.75±3.17^a,b,c^	261.503	<0.001*
Intact neurons	57.63±6.05^c,d^	56.00±3.55^c,d^	31.08±3.45^a,b,d^	39.88±5.57^a,b,c^	69.503	<0.001*

* The difference is statistically significant;

a the difference from the Control Group was statistically significant;

b the difference from the adrenomedullin (ADM) Group was statistically significant;

c the difference from the ischemia/reperfusion (I/R) Group was statistically significant;

d the difference from the I/R+ADM Group was statistically significant. Different letters denote significant differences (*P*<0,05).

SD=standard deviation

## DİSCUSSİON

Cerebral İ/R injury is a clinical picture of progressive brain cell injury caused by İ/R after cerebral ischemia. In the course of I/R process, necrosis and apoptosis may occur during neuronal death^[18]^. Although the reason for I/R injury is not fully explained, there are many different pathological mechanisms. Necrosis, oxidative stress, apoptosis, calcium deregulation, adenosine triphosphate depletion, and excitotoxicity are defined as mechanisms contributing to cerebral I/R injury^[19]^. Cerebral I/R injury is caused by reperfusion of oxygenated blood after ischemia by creating excess ROS in cerebral tissue. ROS is a strong oxidizing and reducing agent that causes cell membrane injury by lipid peroxidation, resulting in neutrophil activation^[20]^. In biochemical events activated by cerebral I/R injury, loss of tissue energy supply occurs firstly, and consequently, endothelial dysfunction and neutrophil sequestration are followed by ROS formation^[21,22]^. Therefore, any factor that suppresses these formations can be applied for the treatment of cerebral ischemia. Cerebral I/R injury studies have been performed previously with bilateral carotide artery occlusion modeling, but there is still no treatment that completely eliminates this injury.

In this experimental study, the protective efficacy of ADM on cerebral I/R injury, which shows some properties such as antioxidative and apoptosis suppressor, was investigated^[23,24]^.

In our study, the antioxidant activity of ADM was evaluated by considering MDA, SOD, GPx, and IMA in brain tissue and blood serum following cerebral I/R. Measurement of antioxidative and oxidative changes can provide indirect information about enzyme activity and the number of ROS. Polyunsaturated fatty acid residues in cell membranes are highly sensitive to oxidation. Lipid peroxidation of cell membranes can lead to cell death during I/R injury. MDA is a biochemical indicator that is indicative of lipid peroxidation. In an experimental study conducted in rats by Oyar EO et al.^[24]^, it was showed increased renal tissue MDA levels after renal I/R injury and significantly lower renal tissue MDA levels in the ADM group after I/R. In our study, there was an insignificant decrease in the elevated tissue MDA levels of I/R Group, after ADM administration to I/R+ADM Group. Lack of significance may be due to the small number of rats in the groups. Thus, we suggest that the decrease in the number of rats may induce significance.

In a study by Oktar et al.^[25]^, total antioxidant status and total oxidant status (TOS) values were examined in order to investigate the effect of ADM on distant organ (lung) injury in rats after lower extremity I/R. In the study, it was seen that TOS value, which is an indicator of all oxidant substances, was significantly higher in I/R groups. It has been shown that the value of TOS in the ADM group after I/R decreases significantly. In our study, it was observed that the MDA value showing oxidation increased after I/R, and that this value decreased significantly after I/R by giving ADM. In our study, in accordance with other findings in the literature, it was concluded that ADM had a protective effect against cerebral I/R injuy because increased MDA values after I/R were significantly decreased after ADM administration. These results support the hypothesis that ADM can reduce oxidative stress.

SOD converts reactive hydroxyl radicals, or OH, to hydrogen peroxide and oxygen^[26]^. Glutathione is converted to oxidized glutathione with GPx in the rendering of GPx hydrogen peroxide. Glutathione plays an important role in the severity of cellular injury. GPx protects the tissues against free radicals by converting hydrogen peroxide into harmless by-products^[26,27]^. SOD and GPx are important components of antioxidant defense for organisms. In our study, it was seen that although the brain tissue SOD and GPx values were not statistically significant in I/R Group compared to the Control Group, the enzyme activity was significantly suppressed. Low levels of antioxidant enzymes after I/R suggest that antioxidant enzyme activity may probably be suppressed due to intense oxidative stress. In I/R+ADM Group, I/R-suppressed enzyme activity increased statistically. In this group, the statistically significant increase in I/R-suppressed enzyme activity indicates that ADM increases antioxidative activity in tissues. Strong antioxidative efficacy of ADM has been demonstrated by Kirisci et al.^[27]^ in a skeletal muscle I/R injury study. In their study, it was shown that after ADM administration there was a significant decrease in increased hypoxia inducible factor 1 alpha, or HIF-1 alpha, and MDA values, which are post-I/R oxidation indicators. Again, in this study, unlike our study, it was seen that there was a significant increase in SOD values after I/R and a decrease in antioxidant enzymes by ADM administration. These results have been interpreted as that ADM showed an antioxidative activity and reduced the need for SOD enzyme activity. When our study was evaluated according to these results, it showed that ADM has a protective effect against oxidative agents by activating antioxidant enzymes against cerebral I/R injury. Considering the studies in the literature, the low SOD values after I/R suggest that antioxidant enzyme activity is suppressed probably due to intensive oxidative stress. İn the studies of Takhtfooladi et al.^[28]^ with tramadol, distant organ (heart tissue) injury was investigated in rats by performing lower extremity I/R. As a result of their study, similarly to our study, it was seen that SOD and GPx values decreased with I/R and significantly increased in the group with tramadol and I/R application. Again, in the experimental myocardial I/R injury study performed by Bansal et al.^[29]^ with lycopene in rats, increased GPx activity with lycopene after I/R had similar results to our study. It has been shown that these values have the similar antioxidative activity with ADM. The lack of change in serum antioxidant values after the study suggests that I/R injury is at the tissue level. No change in serum antioxidant values after the study compared to the Control Group points to that I/R injury is at tissue level.

IMA, named albumin cobalt binding test, or ACB test, is a biomarker used in cardiac ischemia, skeletal muscle ischemia, and cerebral ischemia. Modifications that change the binding capacity of albumin to cobalt during I/R may occur with the formation of acidosis and free oxygen radicals^[29,30]^. When we examined serum IMA values, there was a statistically significant increase in I/R Group compared to Control Group. This increase may have been affected by I/R damage. ADM and I/R Groups did not show any change. According to these results, ADM did not change serum IMA values.

Brüning et al.^[31]^ have investigated the diphenyl diselenide in a bilateral carotid I/R study in rats and evaluated interleukin (IL)-1, IL-6, tumor necrosis factor alpha, and interferon gamma with oxidative and antioxidant parameters. After 20 minutes of ischemia and 30 minutes of reperfusion, the rats were sacrificed and samples were collected. It was observed that MDA values were increased in the I/R group, but decreased significantly in the diphenyl diselenide group during the I/R. SOD values and proinflammatory cytokines were increased in I/R group, and decreased significantly in the diphenyl diselenide group during I/R. Consistent with this study, we found that MDA values were significantly increased in I/R Group compared to Control Group and ADM Group. MDA values of I/R+ADM Group was lower than I/R Group, but it was not statistically significant.

In the study of Quartu et al.^[32]^, bilateral carotid I/R was applied - 20 minutes of ischemia followed by 30 minutes of reperfusion. The protective effect of *Pistacia lentiscus L.* essential oil (EO), which has strong anti-inflammatory activity, was investigated. Plasma cyclooxygenase-2 (COX-2) was increased in I/R group, and COX-2 was decreased significantly in EO-administered I/R group. Histopathologically, they showed that dicycloxaenoic acid, which is sensitive to oxidation, was decreased in the frontal cortex of I/R group. Pre-treatment with EO has been shown to prevent this change. In comparison with our study, we found that ADM significantly reduced the picnotic cell and gliosis in brain tissue in the I/R Group, and significantly increased the number of cerebral neurons.

When the groups were examined histopathologically, it was seen that shrinkage in basophilic neurons with picnotic nucleus and gliosis formation in multiple focal areas were observed depending on oxidative agents after İ/R. The cresyl violet is a well-known histological method used to detect cell damage in the central nervous system^[13,33]^. The number of intact neuronal cells decreased significantly in I/R Group compared to the Control Group, which was statistically higher when ADM was given to I/R+ADM Group and this demonstrates its neuroprotective activity. In the lower extremity skeletal muscle İ/R study of Kirisci et al.^[27]^, it was demonstrated that ADM inhibits apoptosis in muscle tissue, decreases lipid peroxidation and oxidative stress, and has cytoprotective activity on skeletal muscle cells. In the study of renal I/R injury in rats performed by Nishimatsu et al.^[34]^, cytoprotective activity of ADM in renal tissue examination was demonstrated. Our study, in accordance with the literature, also demonstrates the neuroprotective activity of ADM histopathologically against cerebral I/R injury.

### Limitations

We have some limitations in this study. The present study included *in vivo* animal model, which may not ideally represent the human metabolism. The lack of apoptosis assessment in the cerebral tissue specimens and advanced biochemical parameters are other main weaknesses of this study.

## CONCLUSİON

In this experimental study, the protective effect of ADM in the cerebral İ/R injury model, which was created by bilateral carotid artery occlusion, was shown both biochemically and histopathologically. In conclusion, it is understood that ADM is an antioxidant.

**Table:** 

Authors' roles & responsibilities	
MK	Substantial contributions to the conception or design of the work; or the acquisition, analysis, or interpretation of data for the work; drafting the work or revising it critically for important intellectual content; final approval of the version to be published; agreement to be accountable for all aspects of the work in ensuring that questions related to the accuracy or integrity of any part of the work are appropriately investigated and resolved
HG	Drafting the work or revising it critically for important intellectual content; final approval of the version to be published; agreement to be accountable for all aspects of the work in ensuring that questions related to the accuracy or integrity of any part of the work are appropriately investigated and resolved
AK	Drafting the work or revising it critically for important intellectual content; final approval of the version to be published; agreement to be accountable for all aspects of the work in ensuring that questions related to the accuracy or integrity of any part of the work are appropriately investigated and resolved
TOM	Substantial contributions to the conception or design of the work; or the acquisition, analysis, or interpretation of data for the work; final approval of the version to be published; agreement to be accountable for all aspects of the work in ensuring that questions related to the accuracy or integrity of any part of the work are appropriately investigated and resolved
DAA	Drafting the work or revising it critically for important intellectual content; final approval of the version to be published; agreement to be accountable for all aspects of the work in ensuring that questions related to the accuracy or integrity of any part of the work are appropriately investigated and resolved
MS	Substantial contributions to the conception or design of the work; or the acquisition, analysis, or interpretation of data for the work; final approval of the version to be published; agreement to be accountable for all aspects of the work in ensuring that questions related to the accuracy or integrity of any part of the work are appropriately investigated and resolved
AD	Substantial contributions to the conception or design of the work; or the acquisition, analysis, or interpretation of data for the work; final approval of the version to be published; agreement to be accountable for all aspects of the work in ensuring that questions related to the accuracy or integrity of any part of the work are appropriately investigated and resolved
GB	Substantial contributions to the conception or design of the work; or the acquisition, analysis, or interpretation of data for the work; final approval of the version to be published; agreement to be accountable for all aspects of the work in ensuring that questions related to the accuracy or integrity of any part of the work are appropriately investigated and resolved
EA	Substantial contributions to the conception or design of the work; or the acquisition, analysis, or interpretation of data for the work; final approval of the version to be published; agreement to be accountable for all aspects of the work in ensuring that questions related to the accuracy or integrity of any part of the work are appropriately investigated and resolved
